# Prevalence of *Helicobacter pylori* Infection in Peptic Ulcer Patients of Highly Endemic Kashmir Valley

**DOI:** 10.1155/DTE.6.31

**Published:** 1999

**Authors:** Gh. Jeelani Romshoo, G. M. Malik, J. A. Basu, M. Yousuf Bhat, A. R. Khan

**Affiliations:** 1 Department of Gastroenterology SMHS Hospital Government Medical College Srinagar Kashmir India; 2 Department of Medicine SMHS Hospital Government Medical College Srinagar Kashmir India; 3 Department of Pathology SMHS Hospital Government Medical College Srinagar Kashmir India; 4 C/O: Post Box No. 757 G.P.O. Srinagar Kashmir 190001 India

## Abstract

*Objective* This study aimed to find out prevalence of *Helicobacter pylori* (*H. pylori*) in peptic ulcer disease (PUD) which is highly endemic disease in Kashmir.

*Method* This study consisted of 50 PUD patients and 30 asymptomatic volunteers. Peptic ulcer was diagnosed by endoscopic examination and *H. pylori* was detected by histology (using Giemsa stain), one minute endoscopy room test (OMERT) and modified Gram's staining. Positive results from OMERT plus histology were considered as the “*gold standard*” for the presence of *H. pylori*.

*Results* Out of 50 patients, 46 had duodenal ulcer (DU), 2 had benign gastric ulcer (GU) and 2 had both DU and GU. The sensitivity and specificity of OMERT were 94% and 96.70%, histology 97.90% and 96.90% and Gram's staining 91.30% and 85.30%, respectively, as compared to our gold standards. *H. pylori* was present in 76.09% of DU, 50% of GU, whereas patients with duodenitis, channel ulcers, chronic active DU and those with multiple ulcers were 100% *H. pylori* positive. *H. pylori* was present in 10 (33.33%) of healthy volunteers.

*Conclusion* A significant association between *H. pylori* infection and PUD was found in this study. However, there seem to be other causative factors as well which contribute for this highly endemic disease.


*Diagnostic and Therapeutic Endoscopy*, Vol. 6, pp. 31–36
Reprints available directly from the publisher
Photocopying permitted by license only

© 1999 OPA (Overseas Publishers Association) N.V.
Published by license under
the Harwood Academic Publishers imprint,
part of The Gordon and Breach Publishing Group.
Printed in Malaysia.

### Prevalence of *Helicobacter pylori* Infection in Peptic Ulcer Patients of Highly Endemic Kashmir Valley – A Preliminary Study

GH. JEELANI ROMSHOO[a,*], G.M. MALIK[a], J.A. BASU[a],
M. YOUSUF BHAT[b] and A.R. KHAN[c]

[a]*Department of Gastroenterology*, [b]*Department of Medicine*, [c]*Department of Pathology, SMHS Hospital, Government Medical College, Srinagar, Kashmir, India*

*(Received 9 March 1999; Revised 14 May 1999; In final form 21 June 1999)*

***Objective*** **This study aimed to find out prevalence of *Helicobacter pylori* (*H. pylori*) in peptic ulcer disease (PUD) which is highly endemic disease in Kashmir.**

***Method*** **This study consisted of 50 PUD patients and 30 asymptomatic volunteers. Peptic ulcer was diagnosed by endoscopic examination and *H. pylori* was detected by histology (using Giemsa stain), one minute endoscopy room test (OMERT) and modified Gram's staining. Positive results from OMERT plus histology were considered as the "*gold standard*" for the presence of *H. pylori.***

***Results*** **Out of 50 patients, 46 had duodenal ulcer (DU), 2 had benign gastric ulcer (GU) and 2 had both DU and GU. The sensitivity and specificity of OMERT were 94% and 96.70%, histology 97.90% and 96.90% and Gram's staining 91.30% and 85.30%, respectively, as compared to our gold standards. *H. pylori* was present in 76.09% of DU, 50% of GU, whereas patients with duodenitis, channel ulcers, chronic active DU and those with multiple ulcers were 100% *H. pylori* positive. *H. pylori* was present in 10 (33.33%) of healthy volunteers.**

***Conclusion*** **A significant association between *H. pylori* infection and PUD was found in this study. However, there seem to be other causative factors as well which contribute for this highly endemic disease.**

*Keywords:* Giemsa stain, *Helicobacter pylori*, Peptic ulcer, Volunteers

### INTRODUCTION

Kashmir valley is a highly endemic region for peptic ulcer disease (PUD) in North West India [1] having a point prevalence of PUD as 4.72% and a life time prevalence of 11.22% [2]. This is very high compared to the prevalence rate of 1.04 in general population of India, and a life time prevalence of 0.61% in Delhi, 0.69% in Chandigarh and 0.75% in Madras (now Chennai) [3–5].

* Corresponding author. C/O: Post Box No. 757, G.P.O.: Srinagar, Kashmir 190 001, India. Tel.: 01932-34293.

PUD is a multifactorial disorder resulting from imbalance between various aggressive (acid, pepsin) and defensive factors (mucus, bicarbonate, prostaglandins, cell regeneration and blood flow). Besides, antral infection with a Gram negative, 's' shaped, catalase and oxidase positive, microaerophilic, multiflagellate *Helicobacter pylori* (*H. pylori*) organism has also been reported to play an important role in the pathogenesis of PUD [6–9].

*H. pylori* though a non-invasive organism damages the gastric mucosa by secreting various toxins and enzymes which cause local mucosal damage ("leaking roof" hypothesis) [10]. *H. pylori* also causes destruction of antral D cells which are sources of somatostatin (inhibitor for gastrin release). Somatostain inhibition causes increase in gastrin release and hence increases gastric acidity, thereby leading to gastroduodenal injury ("Gastrin Link" Hypothesis) [11]. Other bacterial substances like urease, catalase, mucinase, lipase, hemolysins, phospholipase A, leucotriene-B4, interleukin-1,4,6 and platelet activating factors have been proposed to contribute in the pathogenesis of *H. pylori* induced PUDs and other *H. pylori* related diseases like gastritis, malignancies of stomach viz. adenocarcinoma and non-Hodgkins lymphoma of mucosa associated lymphoid tissue (MALT) [12,13].

This is the first preliminary study from the Kashmir valley which has been carried out to find the association of *H. pylori* organism with the highly endemic PUD. This study also aimed to find the prevalence of *H. pylori* infection in the asymptomatic healthy volunteers of the region.

### PATIENTS AND METHODS

This study comprised 50 PUD patients who had symptoms of dyspepsia and in whom diagnosis of the disease was made by upper GI endoscopy. It also included 30 asymptomatic healthy volunteers. The endoscopy was performed in the endoscopy section of SMHS Hospital of Government Medical College, Srinagar, using an Olympus GIF-GQ-Fiberoptic gastroduodenoscope. Sedation (Inj. diazepam) was given only to apprehensive patients. Patients were endoscoped after overnight fasting of 12 h. Peptic ulcers were assessed by their location, shape, size, number, degree of scarring and deformity of the bulb. Endoscopically an ulcer was classified as acute ulcer – when an area of denuded epithelium of $> 0.5$ cm with a definite depth and with or without slough at the base was present, chronic ulcer – an ulcer with or without slough at the base with scarring and deformity, healed ulcer – when endoscopic examination revealed only scar with or without deformity. The ulcer size was determined using standard gastric biopsy forceps as guidance. The forceps measured 6 mm between the tips in open position. Apparent depth of $> 1$ mm of ulcer was based on experienced endoscopists' judgement.

Four antral biopsies were taken in all subjects for identification of *H. pylori* by different methods viz. one minute endoscopy room test (OMERT) (CLO or urease test), Gram's staining and histopathological examination using Giemsa stain. In cases of gastric ulcer (GU), an additional biopsy was taken from the edge of the ulcer to exclude its malignant nature. The subjects who gave history of ingestion of antibiotics, $H_2$-blockers, NSAIDS, colloidal bismuth or metro-nidazole/tinidazole one month prior to endoscopy were excluded from this study. Multiple punch biopsies from gastric antrum were also taken in all volunteers. *H. pylori* organism was identified by following three tests:

(1) OMERT (urease test): A freshly prepared 10% w/v urea solution in deionized water at a pH of 6.8 was taken in two 5 ml capacity test tubes. Two drops of freshly prepared 1% phenol was added to each tube. One tube served as a reagent and other as a control. Biopsy material was put into the reagent and change of colour from yellow to pink within 15 min was taken as positive, thereby meaning the presence of *H. pylori* [14–18].
(2) Microbiology: Another biopsy material was rubbed over a clean and dry glass slide and heat-fixed. The tissue was stained with Gram's stain and observed under light microscope for Gram negative spiral shaped *H. pylori* bacilli [7,19].

(3) Histopathology: Paraffin embedded 3–5 μm thickness sections were made and stained with Hematoxylin and Eosin and Giemsa stain separately to study histopathological features of gastric mucosa (i.e. type of gastritis) and for small curved *H. pylori* bacilli, respectively [14–18]. Positive results from combined histology plus OMERT were considered as "gold standard" for the presence of *H. pylori* infection [20–24].

(4) Statistical Analysis: Chi-square test was used to analyse the results. A $P$-value of less than 0.05 was considered to be significant.

(5) Ethics: Both patients as well as volunteers gave informed consent for performing endoscopy and for obtaining biopsy tissue. Human experimentation guidelines laid by "Decleration of Helsinki" were followed and this study was approved by Principal/Dean, Government Medical College, Srinagar, after consideration and approval by members of board of studies.

### RESULTS

This study comparised, 50 PUD patients (42 males, 8 females) and 30 healthy asymptomatic volunteers (25 males, 5 females) in the age group of 18–70 years (Table I). Among the 50 PUD patients, 46 had duodenal ulcer (DU), 2 had GU whereas 2 had both GU as well as DU. Epigastric pain was present in 84%, post-prandial fullness in 68%, melena in 60%, hematemesis in 40%, repeated vomiting in 24% and indigestion in 16% of peptic ulcer cases. Volunteers were free of any symptoms. The various endoscopic findings observed in PUD patients are depicted in Table II (many patients had more than one endoscopic lesion). *H. pylori* positivity among cases and controls by three different test methods is shown in Fig. 1. Of the 46 DU patients, 35 (76.09%) were positive for *H. pylori* infection by both OMERT plus histology (Giemsa stain) whereas out of two benign GUs only one (50%) patient was positive for *H. pylori* by these test methods. However, patients with duodenitis, active DU, combined GU and DU were 100% positive for *H. pylori* infection (Table III and Fig. 2). *H. pylori* status had no relationship with sex of an individual but *H. pylori* prevalence increased with the advanced age, and a highest positivity of 90.90% was observed in the age group of 60–69 years. The major blood group among the PUD patients was blood group 'O' (56%) whereas there was no predominant blood group in the volunteers. *H. pylori* status did not show any association with any ABO blood group or rhesus state among both groups ($\chi^2$ycdfl = 5.51, $P > 0.10$ and $\chi^2$ycdfl = 8.61, $P > 0.05$, respectively, in cases and controls).

TABLE I Age and sex characteristics of cases and volunteers

| Group | Cases | Volunteers |
|---|---|---|
| Number | 50 | 30 |
| Age (mean ± SD) | 30.88 ± 12.80 | 29.80 ± 11.22 |
| Sex | | |
| Males | 42 (84.00%) | 25 (83.33%) |
| Females | 08 (16.00%) | 05 (16.67%) |

TABLE II Various endoscopic findings in peptic ulcer patients ($n = 50$)

| Site | Endoscopic features | No and percentage |
|---|---|---|
| Oesophagus | Normal | 44 (88.00) |
| | Reflux oesophagitis | 06 (12.00) |
| Stomach | Antral erosions | 08 (16.00) |
| | Fundal erosions | 01 (02.00) |
| | Antral erythematous gastritis | 30 (60.00) |
| | GU | 04 (08.00) |
| | Outlet obstruction (partial) | 05 (10.00) |
| | Post-operative stomach | 02 (4.00) |
| | Biliary gastritis | 02 (4.00) |
| | Normal | 28 (56.00) |
| Duodenum | Duodenitis | 4 (8.00) |
| | Acute DU | 9 (18.00) |
| | Partially healed ulcer | 28 (56.00) |
| | Deformity of duodenal bulb with bridge formation | 15 (30.00) |
| | More than one DU | 2 (4.00) |
| | Normal | 2 (4.00) |

*Note*: More than one feature was present in many patients. Two patients had both GU as well as DU. Two patients had two separate DUs.

*H. pylori* status did not show any relationship with smoking status in both groups ($\chi^2$ycdfl = 0.830; $P > 0.25$ and $\chi^2$ycdfl = 0.03; $P > 0.75$ among cases and controls, respectively). In PUD patients, antral gastritis changes were observed in 30 patients (60%) on H&E stained antral sections whereas 8 (26.66%) asymptomatic healthy volunteers had evidence of antral gastritis on such examination (Table IV). The sensitivity, specificity and positive and negative predictive values of these three tests when compared to our gold standard are shown in Table V.

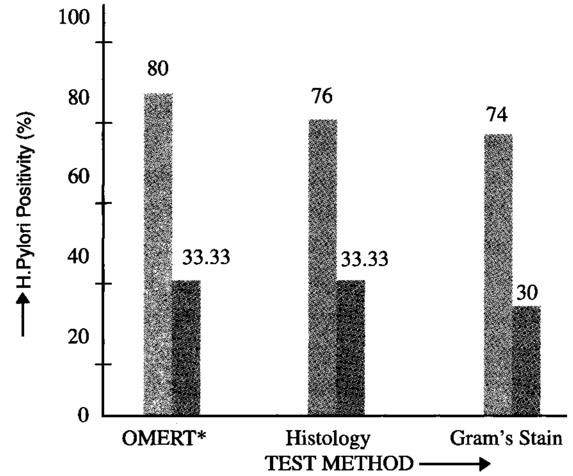

*OMERT = One minute endoscopy room test

FIGURE 1 *H. pylori* positivity by three different tests among cases and controls.

TABLE III *H. pylori* positivity by our gold standard in various endoscopic lesions

| Endoscopic findings | *H. pylori* positivity (%) |
|---|---|
| Acute DU | 88.88 |
| Chronic active DU | 100.00 |
| Combined DU and GU | 100.00 |
| Channel ulcer | 100.00 |
| Two DUs | 100.00 |
| Duodenitis | 100.00 |
| Benign GU | 50.00 |
| Chronic healed DU | 85.71 |

DU = Duodenal ulcer, GU = Gastric ulcer.
*Note*: Many patients had more than one endoscopic lesion.

### DISCUSSION

PUD is a cosmopolitan disease of multifactorial and heterogeneous origin resulting from an imbalance between various aggressive and defensive factors. The different factors important in the pathogenesis

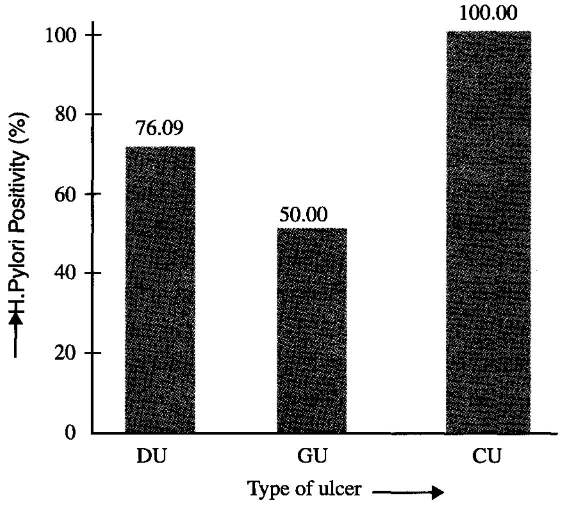

DU = Duodenal ulcer, GU = Gastric ulcer, CU = Combined DU+GU

FIGURE 2 *H. pylori* positivity by our gold standard in different types of peptic ulcers.

TABLE IV Histological findings of Hematoxylin and Eosin stained antral biopsy tissue among cases and controls

| Group | Number | Normal gastric mucosa | CSG* | CAG** |
|---|---|---|---|---|
| Peptic ulcer patients | 50 | 20 (40.00%) | 16 (32%) | 14 (28.00) |
| Controls | 30 | 22 (73.34%) | 04 (13.33%) | 04 (13.33%) |

*CSG = Chronic superficial gastritis; **CAG = Chronic active gastritis.

TABLE V Sensitivity, specificity and positive and negative predictive values of the diagnostic tests as compared to our gold standard

| Test method | Sensitivity (%) | Specificity (%) | PPV** (%) | NPV*** (%) |
|---|---|---|---|---|
| Histology | 97.9 | 96.9 | 97.9 | 96.9 |
| OMERT* | 94 | 96.7 | 97.9 | 90.6 |
| Grams staining | 91.3 | 85.3 | 89.4 | 87.9 |

*OMERT = One minute endoscopy room test; **PPV = Positive predictive value; ***NPV = Negative predictive value.

of PUD include increased parietal cell mass, increased post-prandial gastrin release, increased sensitivity to various secretagogues, rapid gastric emptying, increased duodenal acid load, decreased mucosal resistance, various genetic and environmental factors, and above all antral colonization by *H. pylori* has been strongly associated with PUD especially in recurrent and chronic DUs) [4–9]. The actual mechanism of *H. pylori* induced ulcerogenesis is still not clear but various hypotheses have already been mentioned [8–11] and it is now suggested that Schwartz's old dictum "*no acid-no ulcer*" needs to be replaced by "*No H. pylori-no ulcer*" [25].

Our study did not find any difference in the prevalence of *H. pylori* infection among males and females but the prevalence of both PUD and *H. pylori* infection increased after the fourth decade. This high age specific prevalence of infection favours the influence of socio-economic conditions on the adaptation of H. pylori infection as has been reported by many other studies [26–29].

Our study did not find any difference in prevalence rate of *H. pylori* infection among smokers and non-smokers nor was there any difference among various ABO blood groups and rhesus states in both groups (cases and controls). Thereby suggesting that though smoking and blood group 'O' are risk factors for PUD (GU and DU, respectively), these do not increase the risk for *H. pylori* infection and these are independent risk factors in the pathogenesis of PUD. Similar results have been reported by other studies [30–32].

Antral gastritis was present in 60% of PUD patients compared to 26.66% in healthy volunteers. Chronic superficial gastritis (87.50%) and chronic active gastritis (92.86%) (PUD patients) were positive for *H. pylori* infection ($P < 0.025$). Thus strongly supporting the worldwide literature reports of pathogenic role of H. pylori in aetiology of type B gastritis [6–14,19]. The prevalence of antral gastritis was 26.66% in healthy volunteers (87.50%, positive for *H. pylori*), which is significantly lower than the prevalence of 80% as reported by Misra *et al.* [33], but findings similar to ours have been reported by Prabu *et al.* [34]. Since all these studies are from one country (India) but with different climatic, environmental, dietry, social, economic and ethnic characteristics which could favour the regional variations in prevalence of *H. pylori* infection as has also been reported by Jyotheeswaran *et al.* [35].

Global studies reveal the prevalence of *H. pylori* in DU as 80–100% and in GU as 50–75%, however, in this highly endemic PUD region, this first preliminary study found the prevalence of *H. pylori* to be 76.09% in DU and 50% in GU. This is lower than that of 90–100% reported by different global studies including from India. The lower prevalence rate could be because of different diagnostic criteria used in this study and biopsy material was taken only from one site (gastric antrum). Since Kashmir valley differs from rest of the country in dietry habits (excessive use of salt, spices), climatic, environmental, socio-economic and ethnic characteristics (predominant muslim population), there seem to be some other important ulcerogenic factors as well in the pathogenesis of this endemic disease. An interesting and yet unreported observation we made in this study is that lesions like active DU, channel ulcers, multiple peptic ulcers and duodenitis were 100% positive for *H. pylori* organism. When compared to the prevalence among asymptomatic healthy volunteers, the association of *H. pylori* with PUD was highly significant ($\chi^2$dfl = 14.22 and $P <$ 0.001) (Table VI). The lower prevalence rate of *H. pylori* among the healthy volunteers in this high endemic region of PUD also favours strongly the reports that different virulent and non-virulent strains of *H. pylori* exist and that only virulent strains of *H. pylori* are ulcerogenic [36].

In conclusion, although the prevalence of *H. pylori* infection in peptic ulcer patients of this

TABLE VI Association of *H. pylori* infection with PUD

| Group | Number of cases | *H. pylori* positivity | Statistical inference |
|---|---|---|---|
| Peptic ulcer patients | 50 | 38 (76.00%) | $\chi^2$dfl = 14.22 with $P < 0.001$; highly significant |
| Controls | 30 | 10 (33.33%) | |

highly endemic area is highly significant, yet it is not as high as reported from various global studies, therefore there must be some other aetiological factors as well which contribute for the endemicity of PUD in this area. However, eradication of *H. pylori* organism is strongly suggested (preferably after checking *H. pylori* status) in order to reduce the high prevalence of this disease in future.

### References

[1] Durrani, H. Duodenal ulcer profile in a highly endemic area. *JAPI* 1990; **38**(Suppl. I): 692–694.
[2] Khuroo, M.S., Mahajan, R., Zarger, S.A. *et al.* Prevalence of peptic ulcer in India; an endoscopic and epidemiological study in urban Kashmir. *GUT* 1989; **30**: 930–934.
[3] Dogra, J.R. Incidence of peptic ulcer in India with special reference to South India. *Ind. J. Med. Res.* 1941; **29**: 665.
[4] Malhotra, S.L. Peptic ulcer in India and its etiology. *GUT* 1964; **5**: 412–416.
[5] Madangopalan, N., Balakumar, K. and Jaisheeraj, A. Epidemiology of peptic ulcer in India. *Ind. J. Gastroenterol.* 1985; (Suppl): 3–6.
[6] Yamada, T., Ahnen, D., Alpers, D.H. *et al.* Helicobacter pylori in peptic ulcer disease. *JAMA* 1994; **272**: 65–69.
[7] Marshall, B.J. Campylobacter pyloridis gastritis and peptic ulceration. *J. Clin. Pathol.* 1986; **39**: 353–365.
[8] Blaser, M.J. Helicobacter pylori in the pathogenesis of gastroduodenal inflammation. *J. Infect. Dis.* 1990; **161**: 626–633.
[9] Nomura, A., Stemmermann, G.N., Chyou, P.H. *et al.* Helicobacter pylori infection and the risk for duodenal and gastric ulceration. *Ann. Intern. Med.* 1994; **120**: 977–981.
[10] Goodwin, C.S. Duodenal ulcer, campylobacter pylori and the "leaking roof" concept. *Lancet* 1988; **2**: 1967.
[11] Levis Beardshall, K. and Haddad, G. Campylobacter pylori and duodenal ulcer. "The gastrin link". *Lancet* 1989; **1**: 1167.
[12] Peura, D.A. Ulcerogenesis: Integrating the roles of Helicobacter pylori and acid secretion in duodenal ulcer. *Am. J. Gastroenterol.* 1997; **92**: 8S–16S.
[13] Bruce, E.D. Pathogenic mechanisms of Helicobacter pylori. In: *Gastroenterology Clinics of North America*, 1993, Vol. 22: p. 43.
[14] Morris, A., Ali, M.R. and Brown, P. Campylobacter pylori infection in biopsy specimens of gastric antrum: Laboratory diagnosis and estimation of sampling error. *J. Clin. Pathol.* 1989; **42**: 727–735.
[15] Barthel, J.S. and Evertt, E.D. Diagnosis of campylobacter pylori infection. The "Gold standard" and the alternatives. *Rev. Infect. Dis.* 1990; **12**(Suppl): 5107.
[16] Thillaiayagam, A.V., Arvind, A.S. and Cook, R.S. Diagnostic efficiency of an ultrarapid endoscopy room test for Helicobacter pylori. *GUT* 1991; **32**: 467–472.
[17] Hazell, S.L., Borody, T.J., Gal, A. *et al.* Campylobacter pylori gastritis, detection of urease as a marker of bacterial colonization and gastritis. *Am. J. Gastroenterol.* 1987; **82**: 292–296.
[18] Ruiz, B., Janneg, A., Diavolitsis, S. *et al.* One minute test for campylobacter pylori. *Am. J. Gastroenterol.* 1989; **84**: 202.
[19] Jiang, S.J., Liu, W.Z., Zhang, D.Z. *et al.* Campylobacter like organisms in chronic gastritis, peptic ulcer and gastric carcinoma. *Scand. J. Gastroenterol.* 1987; **22**: 553–558.
[20] Deltenre, M., Glupczynski, Y., De Prez, C. *et al.* The reliability of urease tests, histology and culture in the diagnosis of campylobacter pylori infection. *Scand. J. Gastroenterol.* 1989; **24**(Suppl 160): 19–24.
[21] Faigel, D.O., Childs, M., Furth, E.E. *et al.* New non-invasive tests for Helicobacter pylori gastritis: comparison with tissue based gold standard. *Dig. Dis. Sci.* 1996; **41**: 740–748.
[22] Thijs, J.C., Vanzwet, A.A., Thijs, W.J. *et al.* Diagnostic tests for Helicobacter pylori: A prospective evaluation of their accuracy, without selecting a single test as gold standard. *Am. J. Gastroenterol.* 1996; **91**: 2125–2135.
[23] Chodos, J.E., Dworkin, B.M. and Smith, F. Campylobacter pylori and gastroduodenal disease: A prospective endoscopic study and comparison of diagnostic tests. *Am. J. Gastroenterol.* 1988; **83**: 1226.
[24] Mohammad, A.E., Al-Karawi, A., Ali-Jumah, A. *et al.* Helicobacter pylori: Incidence and comparison of three diagnostic methods in 196 Saudi patients with dyspepsia. *Hepatogastroenterol.* 1994; **41**: 48–50.
[25] Isenberg, J.I., Spector, H., Hootkin, L.A. *et al.* An apparent exception to Schwartz's dictum, "no acid-no ulcer". *N. Engl. J. Med.* 1971; **286**: 620.
[26] Abdelrahman E.L., Sheikh Mohammad, Mohammad Ali, Al-Karawi, Abdel Rehman, Abdullah Al-Jumah *et al.* Helicobacter pylori: Prevalence in 352 consecutive patients with dyspepsia. *Annals of Saudi Medicine* 1994; **2**: 134–135.
[27] Graham, D.Y., Adam, E., Reddy, G.T. *et al.* Seroepidemiology of Helicobacter pylori infection in India: Comparison of developing and developed countries. *Dig. Dis. Sci.* 1991; **36**: 1084–1088.
[28] Katelaris, P.H., Tippett, G., Brennan, R. *et al.* Prevalence of Helicobacter pylori and peptic ulcer and relation to symptoms in a Tibetan refugee population in Southern India. *GUT* 1991; **32**: A556 (Abstract).
[29] Khan, A.R. An age and gender-specific analysis of Helicobacter pylori infection. *Annals of Saudi Medicine* 1997; **18**: 6–8.
[30] Umlauft, F., Keeffe, E.B., Offner, F. *et al.* Helicobacter pylori infection and blood group antigens: Lack of clinical association. *Am. J. Gastroenterol.* 1996; **91**: 2135–8.
[31] Loffeld, R.J.L.F. and Stobberingh, E. Helicobacter pylori and ABO blood groups. *J. Clin. Pathol.* 1991; **44**: 516–517.
[32] Maxton, D.G., Srivastava, E.D. and Whorwell, P.J. Do non-steroidal anti-inflammatory drugs and smoking predispose to Helicobacter pylori infection? *Postgrad. Med. J.* 1990; **66**: 717.
[33] Misra, V., Misra, S., Dwivedi, M. *et al.* Point prevalence of peptic ulcer and gastric histology in healthy Indians with Helicobacter pylori infection. *Am. J. Gastroenterol.* 1997; **92**: 1487–1491.
[34] Probhu, S.R., Ranganathan, S. and Amrapurkar, D.N. Helicobacter pylori in normal gastric mucosa. *J. Assoc. Physicians India* 1994; **42**: 863–864.
[35] Jyotheeswaran, S., Shah, A.N., Jin, H.O. *et al.* Prevalence of Helicobacter pylori in peptic ulcer patients in Greater Rochester, NY: Is empirical triple therapy justified? *Am. J. Gastroenterol.* 1998, **93**: 574–578.
[36] Weel, J.F.L., Vand der Hulst, R.W.M., Gerritis, Y. *et al.* The interrelationship between cytotoxin association gene A, vacuolating cytotoxin and Helicobacter pylori related diseases *J. infect. Dis.* 1996; **173**: 1171–1175.

